# Scaling up complex interventions: insights from a realist synthesis

**DOI:** 10.1186/s12961-016-0158-4

**Published:** 2016-12-19

**Authors:** Cameron D. Willis, Barbara L. Riley, Lisa Stockton, Aneta Abramowicz, Dana Zummach, Geoff Wong, Kerry L. Robinson, Allan Best

**Affiliations:** 1 Propel Centre for Population Health Impact, Faculty of Applied Health Sciences, University of Waterloo, Waterloo, ON Canada; 2 Menzies Centre for Health Policy and the Australian Prevention Partnership Centre, University of Sydney, Sydney, NSW Australia; 3 Nuffield Department of Primary Care Health Sciences, University of Oxford, Oxford, United Kingdom; 4 Centre for Chronic Disease Prevention, Public Health Agency of Canada, Ottawa, ON Canada; 5 InSource Research Group, West Vancouver, BC Canada

**Keywords:** Scaling up, Complex interventions, Chronic disease prevention, Public health, Population health

## Abstract

Preventing chronic diseases, such as cancer, cardiovascular disease and diabetes, requires complex interventions, involving multi-component and multi-level efforts that are tailored to the contexts in which they are delivered. Despite an increasing number of complex interventions in public health, many fail to be ‘scaled up’. This study aimed to increase understanding of how and under what conditions complex public health interventions may be scaled up to benefit more people and populations.

A realist synthesis was conducted and discussed at an in-person workshop involving practitioners responsible for scaling up activities. Realist approaches view causality through the linkages between changes in contexts (C) that activate mechanisms (M), leading to specific outcomes (O) (CMO configurations). To focus this review, three cases of complex interventions that had been successfully scaled up were included: Vibrant Communities, Youth Build USA and Pathways to Education. A search strategy of published and grey literature related to each case was developed, involving searches of relevant databases and nominations from experts. Data extracted from included documents were classified according to CMO configurations within strategic themes. Findings were compared and contrasted with guidance from diffusion theory, and interpreted with knowledge users to identify practical implications and potential directions for future research.

Four core mechanisms were identified, namely awareness, commitment, confidence and trust. These mechanisms were activated within two broad scaling up strategies, those of renewing and regenerating, and documenting success. Within each strategy, specific actions to change contexts included building partnerships, conducting evaluations, engaging political support and adapting funding models. These modified contexts triggered the identified mechanisms, leading to a range of scaling up outcomes, such as commitment of new communities, changes in relevant legislation, or agreements with new funding partners.

This synthesis applies and advances theory, realist methods and the practice of scaling up complex interventions. Practitioners may benefit from a number of coordinated efforts, including conducting or commissioning evaluations at strategic moments, mobilising local and political support through relevant partnerships, and promoting ongoing knowledge exchange in peer learning networks. Action research studies guided by these findings, and studies on knowledge translation for realist syntheses are promising future directions.

**Electronic supplementary material:**

The online version of this article (doi:10.1186/s12961-016-0158-4) contains supplementary material, which is available to authorized users.

## Background

Over recent years, the field of implementation science has garnered much interest as a way to study and understand how to close the gap between what we know and what we do to optimise the health of populations [1, 2]. This interest has stemmed from an increasing recognition that, despite the development of innovative products, practices and programs, we have often fallen short of realising their full potential impact, due to widespread adoption and scaling up challenges [3]. Literature and research on scaling up remains relatively new, and dominated by a focus on single or ‘simple’ interventions [4, 5]. The result is limited knowledge of how to increase the implementation and sustainability of complex interventions that are often used to address population health and other social issues [4]. This study responds to calls from researchers and those involved in designing or delivering complex interventions, to understand how, why and under what conditions such interventions may be scaled up in order to meet the needs of the populations they serve.

Various definitions exist in the literature on what constitutes ‘scaling up’. The concept is often considered to refer to a series of processes to introduce innovations with demonstrated effectiveness through a programme delivery structure with the aim of improving coverage and equitable access to the innovation(s) [3]. As noted by Mangham and Hanson, scaling up is often thought of as a process requiring “*a strategy and implementation plan that considers the policy context, delivery mechanisms and resource requirements, as well as the pace of change, sequencing of activities, areas for prioritization and monitoring and evaluation*” ([3] p. 87). Research on scaling up therefore finds many points of intersection with other implementation-oriented work, particularly that focused on the sustainability of interventions [6–9]. While conceptually distinct, many of the factors influencing sustainability resonate with those also thought to influence scaling up, including characteristics of the innovation or intervention itself, the organisational context, and the capacity (internal and external) and processes related to sustainability and scaling up [8]. Research agendas focused on sustainability are also emerging, calling for greater exploration of key sustainability issues such as methods for sustainability research and infrastructure to support sustainability research [9].

Also consistent with these related domains, much scaling up research to date – particularly in health settings – has focused on understanding the scaling up of discrete and well-bounded interventions such as vaccines and fluoride in drinking water [4, 5, 10, 11]. These studies have yielded important insights into the implementation and adaptive requirements for simple interventions, yet provide little guidance on the challenges of scaling up interventions related to complex social problems such as poverty, the use of illicit substances and the rising prevalence of chronic diseases [12]. As noted by Luke and Stamakis, these problems are characterised by a number of factors, “*they are made up of a large number of heterogeneous elements; these elements interact with each other; the interactions produce an emergent effect that is different from the effects of the individual elements; and this effect persists over time and adapts to changing circumstances*” ([13] p. 358). In response to these problems, many complex public health interventions have been developed, particularly for addressing pressing population health and social issues. Such complex interventions typically include a set of purposefully coordinated components that target multiple levels and sectors of a system [14], that operate both independently and inter-dependently [15], and that interact in the contexts in which they are implemented [16, 17]. Examples of these types of complex interventions include multi-level tobacco control strategies, comprehensive school health initiatives, and community-based poverty reduction activities [18, 19].

Despite the plethora of complex interventions for addressing health and social issues (included under the broad umbrella of public health), there is comparatively little understanding of the actions that can be taken to scale up these interventions in order to extend the breadth and depth of benefit to those in need. Moreover, there is little understanding about how these actions modify the contexts in which they are embedded, which in turn influence the outcomes of scaling up efforts.

This study addresses a major knowledge gap and responds to calls from key policy and practice partners who are responsible for scaling up complex interventions. These partners want to better understand what actions they may use to scale up effective, complex interventions, in what contexts and with what outcomes. Given this focus, this study had the aim of increasing understanding of how and under what conditions complex public health interventions may be scaled up in order to benefit more people and populations.

More specifically, this study addressed the following research question, which explores elements and their relationships central to a realist synthesis: What are the mechanisms by which scaling up outcomes for complex public health interventions are achieved, and what actions can be used to change contexts in order to activate these mechanisms?

## Methods

To address this study’s research questions, a realist synthesis was selected. Realist syntheses aim to ‘unpack’ the mechanisms by which complex interventions work (or the reasons for their failure) in particular circumstances and settings [20]. In doing so, realist syntheses view causality through critical linkages between contexts (e.g. characteristics of subjects and locality), mechanisms (hidden processes or entities – often contained in the minds of people – that are sensitive to variations in context and that generate outcomes), and outcomes (the range of effects that occur over time) [20–22]. Through a realist lens, actions change contexts, activating certain mechanisms leading to specific outcomes. These relationships are specified as context–mechanisms–outcomes (CMO) configurations.

For scaling up complex interventions, relevant actions are those that actors undertake in order to support the scale up of interventions, ultimately to effect positive change in population outcomes. Using insights from selected cases of scaled up interventions (see section on focusing the synthesis), our synthesis generated multiple CMO configurations for each case, which were aggregated across cases according to common mechanisms. We then interpreted these findings in light of relevant theory and practitioner experience.

### A participatory team approach

The synthesis was led by a core team (n = 5) who were guided by a panel of knowledge users (n = 10) and a panel of international experts (n = 7). The purpose of the knowledge user panel was to anchor the synthesis in current practices and related questions for scaling up complex interventions, while the purpose of the panel of international experts was to ensure methodological rigour and consistency with current scientific practice. Given this study’s potential to inform scaling up theory and practices in public health, knowledge user panel members were identified from leading Canadian organisations active in scaling up complex interventions in public health settings. In contrast, members of the panel of international experts were invited with backgrounds in complex interventions, implementation science (including scaling up), realist synthesis methodology and systems change. Both panels were engaged virtually and in-person during the synthesis process, including for refining synthesis questions, focusing the synthesis on a subset of information rich initiatives, developing the literature search strategy and interpreting data.

### Developing the programme theory

The purpose statement and research question for this synthesis were developed in partnership with panellists, and refined following an initial exploration of relevant literature gathered as part of the programme theory development. The programme theory provides an overview of the ‘terrain’ to be investigated, and a structure for organising synthesis findings [22]. As this study did not focus on a specific intervention, the programme theory was generated from diverse evidence related to a variety of complex interventions (as defined above). Full methodological details, including search terms on how the programme theory was developed, are described in Additional file 1. Briefly, an initial search of published and grey literature was conducted, focusing on reviews of scaling up, existing frameworks on scaling up, as well as reported facilitators and barriers to scaling up. The programme theory identified different classifications of scaling up (e.g. horizontal, vertical, functional, etc.), multiple actions for scaling up complex interventions, an initial set of proximal scaling up outcomes, as well as descriptions of potential contextual factors influencing scaling up actions and outcomes (Table 1). However, the reviewed literature did little to describe the relationships that exist between these elements.

**Table 1:** Comparison of outcomes, actions and contexts identified in the program theory and observed in case study documentation

Outcomes, Actions or Contexts	As identified in program theory	As observed in case study documentation
Outcomes^a^	Initiative adapted to context	Initiative adapted to local community needs
	Increased organizational/community capacity and readiness	Increased local organizational capacity
	Finalized scaling up strategy	Intentional and explicit efforts to scale-up through planned processes
	Increased demand for action	Issue prioritized
	Increased participation of communities	Increased community participation
Actions	Develop and adapt funding models and partners involved	Develop mergers between existing organizations with shared objectives
		Adapt funding model to engage community based organizations
		Implement membership fee for peer learning community
		Identify external resources
		Develop competitive funding proposals
	Assess potential readiness and demand for intervention	Conduct needs assessment
	Engage leaders and other stakeholders: develop shared vision; align priorities; recruit champions	In-person engagement sessions for community groups and organizations in peer learning networks
		Engage national leaders in cross jurisdictional summits
		Identify and engage local representatives and leaders
		Identify and engage local champions
		Launch campaigns to secure local political support
	Establish cross-sectoral and jurisdictional partners	Form new partnerships with key funding and social policy organizations
		Develop coalitions of local and national organizations
	Adapt the initiative design to changing contexts	Refine criteria by which communities/sites are selected
		Adapt governance mechanism for initiative
		Wind-up/ descale the initiative
	Develop an action and implementation plan	Develop action plans by founding individuals
		Hold strategic dialogue for developing action plans
	Systematically evaluate implementation and outcomes	Commission external evaluations
		Conduct evaluations of pilot settings
		Conduct interim evaluations
		Conduct end of campaign evaluations
		Conduct data mining to derive maximal learning
		Develop/implement shared measures
		Conduct economic evaluation
		Conduct social return on investment
	Develop, implement and evaluate a knowledge-to-action strategy	Hold information session for community members
		Share promising results from early evaluations through a range of products and mediums
		Disseminate results widely through tailored information packages
		Conduct site visits
		National summits to encourage in-person knowledge exchange
		Develop communities of practice
		Publicly release evaluation results
		Implement learning community for disseminating information
	Create supportive amendments to policy	Identify and engage lobby groups
	Encourage a systems perspective	Not observed in case study documentation
	Integrate change into organizational culture	Not observed in case study documentation
	Provide technical assistance and training to communities	Provide coaching support to communities
		Review existing evidence
		Review best practices
		Advocated on behalf of community
Contexts	Availability of resources: money, training, technical expertise, data systems, effective communication channels, human resources, managerial skills, evaluation capacity, leadership skills, existing relationships	Credibility of organization
		Staff difficulty in implementing action plan
		High turnover of staff
		Scarcity of financial resources
		Lack of staff skills and capacity
		High quality leadership skills
		History of collaboration
		Funding available
		Lack of time among funders
		Staff skilled in relevant activities
	Degree of political support	Uncertain political support
		Strong political support
	Degree to which socio-cultural conditions and climates support scaling up	Persistent sociological problem
		Social and economic hardship
		Stalling of efforts
		Tragedy in community
	Degree of readiness for intervention	Recognized need for change
		Available local funds
		Realistic expectations
	Evidence of impact of the intervention	Uncertainty/variability of intervention effectiveness
		Promising early results
		A history of intervention success
	Degree of consistency in objectives and mandates between stakeholders	Organizational mandate supports solution
		Alignment with user needs
		Unrealistic expectations
		Users value owned interventions
	Degree of interest and demand for interventions (in communities and organizations)	Other comparable interventions
		Unique needs identified
		Growing support for issue
		Interventions and models available
		Interest from funding organizations
		Variability in funding support

^a^Other outcomes were observed in the case studies but not identified in the program theory: launch of spin-off initiatives; shift in emphasis of initiative; legislation enacted; and launch/renewal of initiative

### Focusing the synthesis: identifying case examples for study

The literature on scaling up is extensive and as a result, the synthesis needed to be focused. This is a well-recognised problem for realist synthesis and recommended in the quality standards for this approach [22]. We decided to focus on understanding pathways for scaling up complex interventions using a subset of examples of scaling up. Potential case examples were identified through two sources: nominations from expert and knowledge user panellists and a scan of the 31 documents reviewed as part of developing the programme theory. Both processes identified examples of complex interventions (defined previously) that were scaled up by engaging more communities over time. This process yielded 11 potential case examples from the expert and knowledge user panels, and 12 potential cases from the review of documents included in the programme theory. To determine the suitability of these 23 cases, each was reviewed by the core team using the following inclusion criteria: (1) the case example focused on scaling up complex interventions in any field; (2) the case had been implemented in developed world contexts; and (3) details of scaling up the complex intervention were well documented (so as to be likely to contain sufficient relevant data) in the peer reviewed and/or grey literature. These criteria excluded 12 cases (one due to insufficient documentation and 11 due to non-complex interventions). From the 11 cases meeting inclusion criteria, this synthesis focuses on three that were deemed by panellists as being most information-rich in relation to this study’s research question. These case examples are Vibrant Communities (VC), Pathways to Education (PTE), and YouthBuild (YB).

#### Vibrant communities

The VC initiative aims to create and grow a movement of diverse leaders and communities from across Canada who are committed to exploring, challenging and testing ways to unleash the potential of communities to reduce poverty and ensure a good quality of life for all citizens [23]. VC was established in 2002 through a partnership of three national sponsors (Tamarack, the Caledon Institute, and the McConnell Foundation) and a series of local communities across Canada [24]. The principles underlying the VC approach have been informed by evolutions in the design of the initiative, from its initial implementation as Opportunities 2000 (in a single community in Ontario), through to its status as Canada’s largest poverty reduction initiative – now in more than 100 communities across Canada. VC is built around three core components: national sponsors, a pan-Canadian Learning Community, and Trail Builders. The three national sponsors (noted above) each bring a different set of competencies and are responsible for different aspects of advancing the VC agenda [25, 26]. The pan-Canadian Learning Community enables all local and national partners to learn together about the challenges and opportunities of the approach being explored, collectively building knowledge and know-how [25]. Finally, Trail Builders are a series of urban collaboratives coordinating poverty reduction initiatives in their local settings, and which receive additional support to pilot new ideas and to track key lessons of value to the broader VC community [25–29]. More details on the VC initiative can be found at http://www.vibrantcanada.ca/.

#### Pathways to education

Like the VC case example, PTE aims to reduce poverty and its effects; however, it has a specific focus on increasing the high school graduation rate in low-income communities in Canada. The PTE initiative began in one neighbourhood in Toronto in 2001, which was experiencing high rates of crime, youth unemployment and low high school completion rates [30, 31]. The PTE initiative was designed to increase high school graduation rates through a programme involving four key supports, namely academic, social, financial, and one-to-one mentoring support [32]. After securing an initial set of funds for local implementation in Toronto, a range of funding agencies joined the initiative, including non-government organisations and federal and provincial governments. As a result, the PTE programme has expanded from the initial neighbourhood in Toronto to more than 17 communities in Ontario, Quebec, Nova Scotia, Manitoba and British Columbia [32]. Further details about the PTE initiative can be found at http://www.pathwaystoeducation.ca/.

#### YouthBuild

YB aims to capitalise on the intelligence and energy of low-income people in order to rebuild their communities and their lives [33]. YB grew from the early efforts of Dorothy Stoneman, a teacher working in east Harlem in the 1960s and 1970s. At the time, thousands of teenagers in the community were failing to complete school and were becoming involved in a cycle of violence [34]. Youth consultations identified a desire to rebuild run-down houses and other community buildings that had been progressively overtaken by drug dealers [35]. Soon after these consultations, the Youth Action Program was founded, which led to the development of YouthBuild USA [35]. The YB initiative engages low-income young people in a 10-month programme, with time spent in academic settings and hands-on training building affordable housing and other community assets. From its beginning in one community in East Harlem, the YB initiative now consists of 260 programs in over 46 states that engage approximately 10,000 students each year, with funding from the US Department of Labor, local non-profit organisations, community colleges and public agencies (the initiative also has an international component not reviewed as part of this study) [36]. For more information on the YB initiative see https://www.youthbuild.org/.

**Fig. 1:**
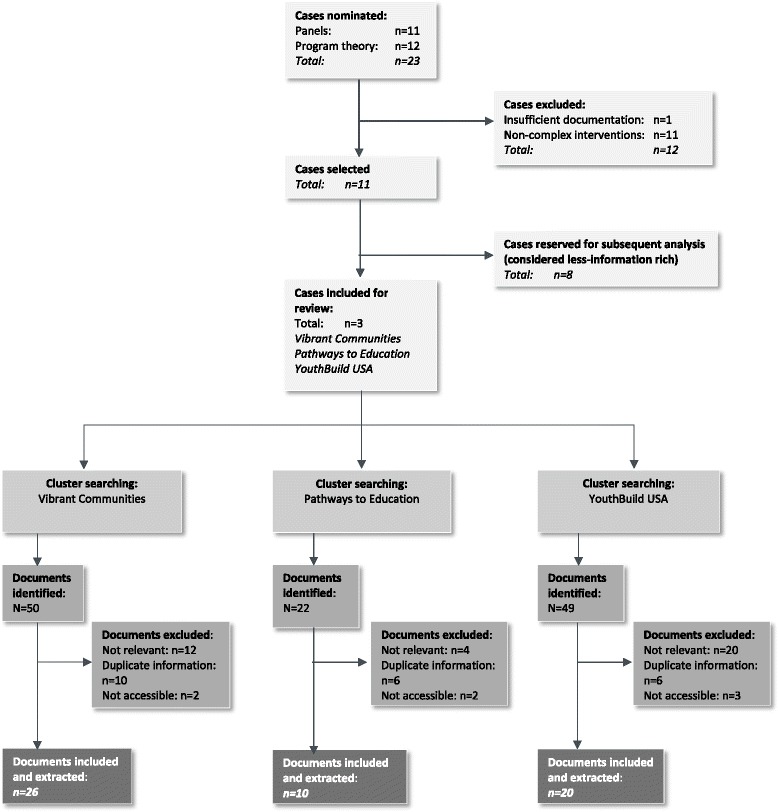
Case and document selection flow chart

Documents related to the above cases were identified through a cluster searching procedure [37]. The initial step was to identify a subset of key documents for each case example (considered as the most comprehensive and recent documents), from which all cited and related references were retrieved, followed by a lead author search for all identified references, and a Google Scholar search for all linked citations. Cluster searching was completed by a single reviewer for each case example. Identified documents were date ordered and screened by two reviewers, beginning with the most recent document. At this stage, documents that (1) did not contain details relevant to the scaling up of the intervention within each case (e.g. solely contained details on impact evaluation); (2) only contained information that was available in a more recently published document; or (3) were not accessible to the core team, were excluded. Disagreements between reviewers were resolved through open discussion. This process resulted in inclusion of 26 documents for the VC case example, 10 documents for PTE, and 20 documents for YB (see Fig. 1 for selection flow chart).

### Data extraction

**Fig. 2:**
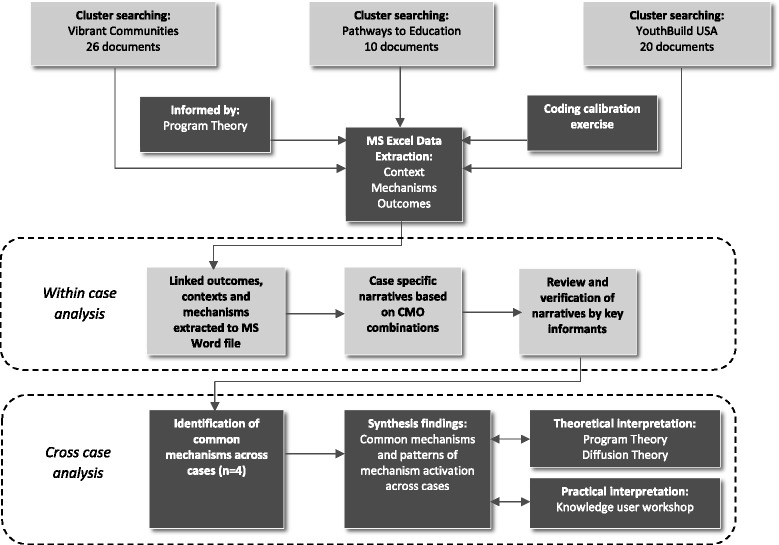
Synthesis process

Figure 2 describes the linked stages and processes involved in extracting and synthesising insights within and across cases. Beginning with the most recently published document, direct text quotations were extracted into an Excel spreadsheet and coded as contextual details (which included how contexts were changed by specific scaling up actions), mechanisms (mostly unstated) or scaling up outcomes. Based on the programme theory and with guidance from the expert panel, contextual details were considered as the characteristics of individuals, organisations, communities or systems which were influenced by deliberate actions or activities by any actor to scale up a complex intervention, mechanisms as the unseen response by people to the specific changes made to the context through actions (e.g. excitement, fear, commitment, etc.), and outcomes as the proximal results of activated mechanisms (e.g. adding new communities, secured financial resources for scaling up, increased knowledge/skills necessary for scaling up, etc.). An initial coding calibration exercise involving five reviewers was completed for four documents, with group discussion to resolve disagreements and support consistency in coding interpretation among reviewers. Following calibration, individual reviewers completed the data extraction for each case example, consulting other members of the core team as needed (e.g. clarifying when data represented a context or an outcome).

### Data synthesis

Data extracted into the Excel spreadsheets for each case were cleaned by two reviewers, with the aim to ensure consistent coding as per C, M and O categories. Within each case, two reviewers identified proximal scaling up outcomes, and the contextual details linked to each outcome. Where stated, the linked mechanisms were also extracted; where the mechanism was not stated, two reviewers agreed on an inferred mechanism. These data were extracted into a Word file as individual CMO configurations. Where outcomes could not be linked to contexts, neither these nor any linked data were extracted. Using the extracted CMO configurations, reviewers created narratives for each case example, which were sent to key informants from each case for verification of accuracy.

As per guidance on realist syntheses [22], we recognised the central role of mechanisms being the causal processes that link contexts to outcomes. As such, we used the identified mechanisms as the focus of the synthesis process across the case examples. Mechanisms identified in only a single case were removed from further analysis, resulting in a set of narratives based around a common set of mechanisms that occurred within more than one of the cases. Two reviewers then examined the activation of these mechanisms across the three cases, noting potentially meaningful patterns of activation. Findings presented from this analysis use CMO configurations from across the case examples to illustrate how different mechanisms may be activated.

### Wider interpretation and links to substantive theory

Findings of realist syntheses are normally interpreted in relation to the programme theory and one or more substantive theories [22]. We interpreted our findings alongside the programme theory, and diffusion of innovation theory, as suggested through consultation with the expert panel [38]. Diffusion of innovation theory relates to the consistent pattern of adoption over time by a population of a new idea or innovation. Diffusion of innovation theory holds that innovations typically demonstrate a slow rate of initial diffusion, followed by a rapid increase in the rate of diffusion as more people and populations adopt it, before a reduction in uptake as laggards eventually adopt the innovation [38]. We used diffusion theory to interpret and extend our understanding of identified CMO configurations and scaling up strategies [38, 39].

To further aid in interpretation of synthesis findings, a one day in-person workshop was held in July 2015 with a mix of 20 national and international research, policy and practice leaders, with experiences in scaling up complex interventions. The purposes of the meeting were to assist in interpreting preliminary study findings, and provide practical input into a research agenda on scaling up complex interventions. Preliminary synthesis results were explored through interactive presentations, small group work and facilitated large group discussions. In concert with connections to relevant theory, insights from the in-person meeting were used to refine synthesis findings and highlight future areas for further inquiry.

## Findings

The following sections present the common mechanisms activated across the three case examples, followed by discussion of their role in scaling up with illustrative CMO configurations from the case examples.

### Scaling up mechanisms

Evidence from the three case examples identified four common mechanisms that appear important for achieving proximal scaling up outcomes for complex interventions: awareness, confidence, commitment and trust. These mechanisms were observed to occur in at least two of the three case examples, at different time points in the scaling up of each intervention, and were activated by different contexts (which were changed through different actions), leading to a range of proximal scaling up outcomes.

#### Awareness

Awareness is considered as knowledge or perception of a situation or fact [40]. In the context of scaling up complex interventions, awareness was interpreted in a number of ways: awareness among community members and local stakeholders that past efforts had reached a plateau of effectiveness or impact; awareness among initiative leaders of the need to adapt scaling up activities to suit their context; or awareness by community leaders that collaborative actions and innovation were required in order to regain momentum.

#### Confidence

Confidence is the feeling or belief that one can rely on someone or something; the state of feeling certain about the truth of something; a sense of self-assurance arising from one’s appreciation of one’s own abilities or qualities [40]. While related to the mechanism of ‘trust’, confidence differs subtly in its more explicit focus on self-assurance. From the data reviewed, we identified confidence in three forms: the confidence of local community members that the initiative was effective for addressing the community’s problem; the confidence of the initiative leaders’ that a particular community had the capacity to implement the initiative; and the confidence among local stakeholders that the leaders of the initiative were capable of successfully implementing the initiative in their settings.

#### Commitment

Commitment is the state or quality of being dedicated to a cause or activity [40, 41]. For scaling up complex interventions, commitment is required from a range of individuals and organisations, including those designing, implementing, evaluating or partnering on an initiative. From the case examples included in this synthesis, commitment was observed to primarily relate to a community’s commitment to being part of or joining an initiative; perceived commitment of an initiative to addressing the needs and concerns of a community; commitment of funders to supporting an initiative; and the commitment of champions (either locally or nationally) to supporting the initiative.

#### Trust

Trust is the firm belief in the reliability, truth, ability or strength of someone or something [40]. As noted, trust is closely related with the mechanisms of confidence and commitment, yet is more of a relational characteristic that was found to precede mechanisms of confidence and commitment. In the included case examples, trust was found to principally relate to the trust among participating organisations (those operating at local and national levels), as well as the degree of trusting relationships between these different levels.

### Activating the identified mechanisms

Insights from the VC, PTE and YB cases help reveal how each of the identified mechanisms may be activated by different changes in context, leading to different outcomes, and at different stages of scaling up. Activation of these mechanisms was observed to occur as part of efforts to renew and regenerate complex interventions, and to document scaling up successes. The following section illustrates different ways in which each of the identified mechanisms may be activated. Relevant text is highlighted as context (C), mechanism (M) or outcome (O).

#### Renew and regenerate

Throughout the multi-year histories of PTE, VC and YB, our analysis identified repeated moments of renewal and regeneration. These moments were signalled by official launches or relaunches of the initiative in a community, regenerated and redesigned initiatives in terms of partners engaged, staff and funding involved, or major transitions for initiatives in their structures, branding or processes. These key inflection points resulted from efforts to design, redesign and re-envisage an initiative, and appear distinct from other (related) activities such as recruitment of individuals, organisations or communities to participate or join an existing initiative, or gradual adaptations made to interventions over time.

While the mechanisms of awareness and commitment were both observed to be activated within efforts to renew and regenerate the YB, PTE and VC cases, this analysis suggests that these mechanisms may be triggered using different actions to change contexts. The following illustrations from PTE and VC highlight specific CMO configurations that reveal the activation of each key mechanism leading to renewal and regeneration of complex interventions. Additional information has been provided for each CMO configuration to enable readers to situate the configurations.

The early stages of the PTE initiative highlight how commitment may be activated to launch a complex intervention. Launching what would become the PTE initiative required the commitment of local leaders and community members to the concept of an intervention in the community. Proponents for an initiative engaged community change experts, helping to identify and articulate the value and design of a “community succession plan”, along with practical ways leaders of the Regent Park Community Health Centre (RPCHC) could participate [31]. These actions took place in a context (C) characterised by longstanding poverty, an increasing rate of high school non-completion, awareness among local stakeholders of the need to break the cycle of poverty, and an innovative board of directors managing the RPCHC [31]. The actions increased the ability of the RPCHC board of directors to recognise the importance of a programme for their community, activating the commitment (M) of the RPCHC, other local leaders and the broader community to a structural change in the centre’s direction. As a result, the ownership of the RPCHC was given to community members [31], allowing local Regent Park residents to lead the identification of obstacles and solutions, and to tailor the initiative to local conditions [31]. After reviewing other programs [42] and widely engaging community members and other stakeholders the “community succession” project was officially launched in 1996 (O) [31].

While commitment of the RPCHC board and other local leaders was the main mechanism for enabling the launch of the PTE in its developmental phase, awareness was identified as a key mechanism for renewing and regenerating the VC initiative, 9 years after its initial implementation.

To ensure continued investment in poverty reduction efforts, VC leaders adopted a broader strategy of renewal and regeneration. Evaluation, including developing expertise in shared measurement approaches, was central to this strategy. End of campaign evaluations were used to raise awareness of the variable experiences of some communities with the VC model, and included data mining to gain maximal insight into the contributing factors influencing community closures and successes (C) [25, 43]. These data helped to raise awareness (M) among national staff of the challenges facing the VC approach, and the need to reconceptualise a national expansion programme. Through greater awareness of what such a national expansion programme may involve, activated by a data-rich context, VC leaders were able to secure transition funding for communities (O) [25], and to continue the engagement of local, regional and national partners (O) [23]. As a result of these activities, the initial VC campaign was wound up in 2011 and re-launched as *Vibrant Communities: Cities Reducing Poverty*, which, as of 2014, included 43 cities and regions that have actively aligned poverty reduction strategies and multi-sector roundtables [25], with plans for an additional 57 communities in 2016 (O) [27].

The examples of PTE and VC provide insights into how commitment and awareness may be activated as part of broader strategies to renew and regenerate complex interventions, in these cases, through effective use of evaluative data (either from other established interventions or a local solution) and engagement with local and inter-jurisdictional partners and community members. Both examples highlight the uncertainty faced by leaders of complex initiatives: in the formative stages of designing an initiative for addressing a problem of local importance or when faced with plateauing effectiveness of an existing intervention. In the PTE example, residents were engaged around expert evidence from external sources to activate local commitment, while in the VC example, the intervention’s own evaluative data were used to raise awareness of the intervention’s successes and challenges, and to use these data to reposition and redesign the approach. In both examples, conditions were characterised by a willingness of organisational leaders to engage around a persistent social and economic problem and to use data to better understand available options.

#### Demonstrate success

The identified mechanisms were also observed to fire as part of efforts to demonstrate the success of the PTE, VC and YB initiatives. In particular, the cases reviewed highlight the role of confidence and trust within broader demonstration efforts, and the cyclical nature of their activation by changes in various contexts (influenced by a range of actions). Our analysis suggests that these mechanisms are key parts of documenting the success of initiatives, which is critical in retaining the participation of communities and recruiting new communities to a scaling up effort.

Despite YB’s 12 years of operation, by 1990, concern existed amongst potential funders that the initiative was unsuitable for scale up due to a potential reliance on the leadership of a single individual [34]. To reduce this concern (and therefore to modify this context), leaders of the YB initiative sought foundation support for implementing a demonstration project outside New York City. This funding was reliant on the development and implementation of an external evaluation and knowledge translation (KT) strategy. Findings from the evaluation and KT activities brought new insights into the process of implementing the YB initiative in sites outside New York City (C), activated confidence (M) among potential funders that investments in the initiative would lead to successful and scalable outcomes (O) [34, 44]. This self-assurance or confidence spurred supporting philanthropic foundations to both use and share knowledge gained from the evaluations to widely disseminate positive messages about the initiative to new communities in new jurisdictions (C) [34]. With the support from the Mott Foundation, Ford Foundation and, later, the DeWitt Wallace Reader’s Digest Fund, the success of demonstration projects allowed the initiative to expand to five additional sites, bringing the total number of sites to 15 by 1993 (O) [34].

In contrast to the YB initiative, insights from the PTE case example highlight how trust may be activated in the early stages of scaling up a complex intervention in new communities [31]. To do so, leaders of the PTE initiative sought to demonstrate the success of the PTE initiative through intensive research and evaluation activities (C). Data from these activities documented the effectiveness of the programme, ensured accountability and programme improvement, and helped identify the replicable aspects of the programme in other communities (C) [45]. To support systematic data collection and sharing, the PTE leadership secured external funding and formed a key partnership with the local school board [45], allowing the collection of baseline data in order to track changes over time (C) [31].

These activities yielded early implementation results of the PTE initiative that demonstrated a reduction in the high school drop-out rate from 56% to 12% [31, 46]. The research and evaluation programme also engaged community members in defining and describing the barriers faced in education and employment, and identifying potential best practices from other programs (C) [31]. These actions were important for activating the trust of community members in the structures, processes and leaders of the PTE initiative (M), leading to their continued support of PTE in their communities (O). The capacity of the PTE initiative to provide support to the Regent Park community over a long period has been highlighted as a key characteristic of effective youth development programs (e.g. Partee GL and Halperin [47]), and noted as a critical vehicle for maintaining continuity in the relationships necessary for effectiveness [31].

The YB and PTE examples illustrate the different ways that commitment and trust may be activated as part of a strategy to document a complex initiative’s success. In both case examples, concern existed among stakeholders (i.e. funders and community members) in the suitability of the intervention for local contexts. The present analysis suggests that, in a long standing initiative (such as YB), an external evaluation and KT plan as part of an explicit demonstration effort, are important actions for activating the commitment of funding agencies. Similarly, the PTE example suggests that, for newly developed initiatives, early evaluation results may be used to activate the trust of community members in the design, implementation and leadership of interventions. In both cases, transparent evaluation and KT actions that engage key stakeholders emerged as essential.

### Links to theory

The synthesis findings were interpreted in light of the original programme theory developed as part of this synthesis, as well as concepts contained in existing frameworks related to scaling up and diffusion of innovation theory.

While the programme theory did not identify specific mechanisms, it did contain relevant contexts and outcomes of scaling up complex interventions. For example, the programme theory identified that the availability of resources was as an important contextual factor for successful scaling up. From the literature reviewed, we found these resources to include financial resources, as well as non-financial resources such as leadership, evidence, relationships and specific skills. These enabling contexts were influenced by a range of actions, including specific efforts to gather useful data from research and evaluation, to develop and adapt funding models and partners, and to engage leaders and other stakeholders (Table 1). As noted in both the programme theory and our findings, these contexts were associated with a range of outcomes, such as increased local organisational capacity, prioritisation of an issue, or recruitment of additional communities. Of note, our findings suggested a number of additionally important outcomes for scaling up complex interventions, such as the launch of spin-off initiatives, or enactment of new legislation.

Findings from the synthesis were largely consistent with recommendations from the small number of scaling up frameworks in the literature, particularly in relation to scaling up actions and important contextual facilitators. As per the case examples reviewed, conducting evaluations was identified across multiple scaling up frameworks as an important facilitator of scaling up efforts [48–50]. In addition, engaging champions and investing in collaborative structures (e.g. networks and partnerships) were also noted to be present in both scaling up frameworks and the literature reviewed in this analysis [48, 50]. Yet, some scaling up frameworks also emphasised a careful, incremental approach to scaling up, which was not consistently evident in the case examples reviewed [49]. This is not to say that planned approaches were not used; simply that the relative emphasis given to this aspect of scaling up was variable across the cases and less than that given to other activities and outcomes such as community engagement and evaluation.

Not surprisingly, the mechanisms identified in the present study (i.e. awareness, commitment, confidence and trust) are not explicitly profiled in scaling up frameworks. However, when interpreted alongside insights from diffusion theory, the synergy of these mechanisms with concepts from diffusion become more apparent. For example, the mechanism of awareness finds support in the ‘problem/opportunity identification’ and ‘communication’ phases of diffusion theory, during which insights from assessments, evaluation and monitoring may be used to raise awareness about the potential impact or utility of an innovation [38]. In contrast, the phase of ‘adoption’ evokes the mechanism of commitment, as opinion leaders begin to demonstrate their support for an innovation in a particular setting. Finally, confidence and trust are woven within and across multiple phases of diffusion theory, as adopters gain experience with an innovation, develop the necessary skills for its implementation, reinvent and renew an innovation, and adapt it to changing conditions and technologies [38, 51].

Findings from this study also suggest potential places where diffusion theory may diverge from the practice of scaling up complex interventions. While greater flexibility has been introduced over time, diffusion theory largely focuses on linear dissemination of an essentially unchanging innovation [38]. In contrast, results from this study highlight importance of renewing and regenerating complex interventions, and of documenting the success of the initiative at key moments. As a result, the complex intervention itself may change over time, in some cases becoming distinct from its initial conceptualisation. This notion of cyclical adaptation and renewal is consistent with other theories of sustaining innovations and implementation science and may warrant further investigation to explore potentially relevant theoretical perspectives [6, 52].

### Interpretation by knowledge users

The data interpretation workshop involving practitioners responsible for scaling up complex interventions identified multiple points of connection between the practice of scaling up and findings from the synthesis. Practitioner experience and the synthesis both noted the value of multi-level partnership and engagement strategies, the importance of leadership and champions, and the utility of evidence and evaluation as scaling up actions to modify contexts. In contrast, workshop participants identified some areas of potential disconnect between the results of the synthesis and their experiences of scaling up complex interventions, such as the limited detail on the multi-level nature of observed mechanisms; the potential differences between urban and rural scaling up settings; and the need to know more about the role of serendipitous events (e.g. unexpected change in staff or team structures) that may help or hinder scaling up efforts.

The data interpretation workshop also identified priorities for a research agenda focused on scaling up complex interventions. Within the strategic areas of renewal and regeneration and documenting success, there are important topics for future exploration, such as how to build and sustain the relationships necessary for scaling up complex interventions; examining the dynamic and multi-level relationships among identified mechanisms; how to foster the necessary skills and other capacities for effective scale up; studying the processes by which interventions and contexts may be adapted over time to optimise ‘fit’; and exploring how evaluation can be used to serve accountability as well as learning and improvement purposes. As noted by participants at the data interpretation workshop, this action-research agenda would benefit from examining scaling up efforts that may have been less successful in order to better understand the nature of supportive or unsupportive conditions for scaling up.

## Discussion

Closing the gap between what we know and what we do requires insights into the nature of interventions and how they interact to influence contexts in which they are embedded [6]. Complex interventions that address challenging health and social issues may target multiple levels in a socio-ecological system (e.g. individuals, organisations, communities and/or broader socio-political/economic/cultural environments) [53]. Ensuring the most people, in the most places, benefit from these interventions requires careful and considerable scaling up effort through deliberate strategies of renewal and regeneration and continually documenting success. Through examining the scaling up experiences of three complex interventions, this study has generated new insights into how specific actions may be used to change context in such a way as to trigger the awareness, confidence, commitment and trust of key stakeholders.

Findings from this work contribute to the theoretical perspectives on scaling up complex interventions and processes for diffusion of innovations, the methods for conducting realist syntheses, and the practical approaches used in scaling up.

This study integrates theoretical concepts from diverse literatures. The interventions reviewed are collaborative in nature, requiring the coordinated action of individuals, organisations, communities and systems. These foci are consistent with contemporary literature on complex interventions in implementation science [15, 54], systems change [55, 56], and inter-organisational collaboration (including partnerships and networks) [57–59]. These findings also resonate with literature on sustainability research, which highlights the role of partnerships in furthering the theory and practice of intervention sustainability [9]. Given this emphasis, it follows that the generative mechanisms identified in this synthesis are largely relational in nature, including concepts of trust, confidence and commitment (noting awareness as a possible exception). Consistent with realist perspectives and literature focused on collaborative action, these mechanisms provide explanations for how specific activities operate on contexts to influence scaling up outcomes. As noted in the illustrative CMO configurations from each case example, each mechanism may be activated in various ways, and by various actors, to achieve a variety of scaling up outcomes such as securing alternative financial resources, expanding the number of communities involved or bolstering political support. These relationships between contexts, mechanisms and outcomes within broader strategies of renewal and regeneration and documenting success provide useful starting points for further examinations using primary data to test and refine emerging theories of scaling up complex interventions.

This study also contributes to the growing use of realist approaches in evidence synthesis. Through guidance from an expert panel, and careful attention to recently published guidelines on realist synthesis, we sought to adhere as closely as possible to the realist synthesis approach. Yet, the consistent classification of contexts (and actions that change them), mechanisms and outcomes remains a challenge, particularly in highly iterative and dynamic fields such as scaling up [60]. For example, given that mechanisms are often confused with the active ingredients of interventions, the expert panel in this study assisted in identifying and applying Jagosh et al.’s definition of a mechanism as “*the cognitive or affective responses of participants to resources offered*” [61]. The participatory nature of our synthesis was therefore an important way for navigating complex definitional terrain, providing regular opportunity for debate, discussion and decision-making within the core team, as well as with regular input from the expert panel. As realist syntheses continue to gain interest and attention, greater sharing of experiences as to how these concepts and decisions are made will be paramount.

Finally, findings from this synthesis may help to advance the practice of scaling up complex interventions. Specific actions appear important for renewing and regenerating complex initiatives, as well as documenting success as a complex intervention is scaled up. These actions include adapting funding models in response to changing resource environments, conducting or commissioning evaluations at different time points throughout scaling up activities, developing and implementing data sharing/feedback processes, identifying and engaging community champions, and building strong foundations of political support. While these (and other) actions are known to practitioners, the present findings link these actions to mechanisms of change (i.e. building awareness, confidence, commitment and trust) and to specific outcomes that may serve as proximal goals for scaling up.

Examining study findings at the data workshop readily surfaced for scaling up practitioners that insights from the synthesis are extensive and nuanced. The relationships between context, mechanisms and outcomes resonated with their experiences and also allowed practitioners to see their experiences in new ways. They especially appreciated the concreteness of actions that can influence context and activate mechanisms, with much less interest in engaging deeply in the latter, which is more theoretical. As noted, the workshop identified some points of disconnect between review findings and practitioner experiences, particularly related to the multi-level nature of mechanisms, rural and urban differences in scaling up strategies, and the role of serendipitous events in enabling or constraining scaling up activities. Rarely are details related to these factors included in documentary evidence, yet workshop participants recognised their importance. One implication for those seeking to better understand scaling up is a more explicit focus on capturing the practitioner wisdom from scaling up experiences and including these insights along with other forms of evidence.

Knowledge user participants at the workshop (many of whom were from public health policy and practice settings), also recognised the need for new approaches to KT that would allow them and their colleagues to apply the insights in their scaling up work. KT approaches would need to set priorities for what findings to share, with whom and when, as well as how. Relevance and timeliness of findings was considered important, and how realist synthesis findings may be tailored for use by different audiences. Informed by knowledge user interests, research is now underway to explore what effective KT strategies for realist synthesis might involve, such as identifying key stakeholders to engage, exploring knowledge requirements, the utility of different modes of delivery, and potential evaluation strategies and feedback designs. The present synthesis provides an initial example for exploring these KT issues in partnership with existing knowledge user and expert panels.

### Strengths and limitations

This study closely followed guidance for conducting realist syntheses, and engaged experts in the methodology. The highly participatory approach to this synthesis enabled broad input and involvement from a variety of knowledge users and experts, increasing the rigour of the synthesis, as well as the utility of findings. However, while focusing on a specific set of cases allowed detailed examination of scaling up, it may have missed important mechanisms evident in other scaling up initiatives.

Under the evidence synthesis funding requirements for this synthesis it was not possible to complete primary data collection as part of this study. As a result, findings from this study are limited to insights drawn from existing documentation relating to each case. The full depth of important contextual details and relationships within and between CMO configurations may therefore be lacking. Exploring these contexts and overall findings from this synthesis through primary data collection is therefore an important next step in advancing this work and our understanding of scaling up complex interventions. The study also focused on successful examples of scaled up interventions, while each contained challenges and barriers to scaling up, future studies investigating less successful scaling up cases may provide important and valuable insights.

## Conclusion

This study responds to calls for understanding and improving scaling up efforts for complex interventions to enhance positive population outcomes. Through in-depth examination of three case examples of successful scaling up of interventions, this synthesis applies and advances relevant theoretical perspectives, contributes to the growing discourse on realist synthesis methodology, and offers insights into how the mechanisms of awareness, commitment, confidence and trust may be activated as part of key strategies to renew and regenerate initiatives and document success when scaling up complex interventions. Findings from this synthesis therefore provide potentially useful directions for future action-research studies to further the science and practice of scaling up.

## Additional file

Additional file 1: Search strategy for developing the programme theory. (DOCX 53 kb)

## Abbreviations

C: context
CMO: context-mechanism-outcome
KT: knowledge translation
M: mechanism
O: outcome
PTE: Pathways to Education
RPCHC: Regent Park Community Health Centre
VC: Vibrant Communities
YB: YouthBuild
