# Supplementary material for: Scaling up complex interventions: insights from a realist synthesis
Source: Health Res Policy Syst. 2016 Dec 19;14:88. doi: 10.1186/s12961-016-0158-4 (PMC5168709; doi:10.1186/s12961-016-0158-4)
Supplement: Additional file 1: — Search strategy for developing the programme theory. (DOCX 53 kb) [file 12961_2016_158_MOESM1_ESM.docx]

**Additional file 1 Search strategy for developing the program theory**

Four iterative searches helped to guide development of the preliminary program theory. The following is an overview of these four searches and the information that was abstracted.

1. **Scale-up reviews**

The purpose of this first search was to identify the most highly relevant reviews on scaling up, and to contextualize the review in light of previous syntheses.

An initial preliminary search was conducted of targeted health and non-health databases: PubMed, EMBASE, and Web of Science using a range of MeSH, Subject Headings and key terms falling into three concepts:

- Concept 1 terms: multi-component/multi-level/complex adaptive systems;
- Concept 2 terms: intervention/program/innovation/best practice/policy/evidence based programs/evidence based practices;
- Concept 3 terms: scaling up, dissemination, implementation, diffusion, scope, reach, adoption, knowledge translation, knowledge transfer, technology change, whole scale change, large scale change, large scale sustainability, widespread sustainability, strategic change, organizational change, system change.

The search was limited to reviews, published in English between 2009-2014 (inclusive).

This process yielded 758 articles (with duplicate articles removed). To inform the development of the program theory, a list of the 100 most relevant reviews (as identified by Web of Science) was used as a starting point. These reviews were examined by two analysts to identify those reviews most relevant to understanding scaling up of complex, multi-component interventions. This process identified 11^1-11^ highly relevant reviews, from which a further bibliographic search identified an additional two^12 13^ relevant reviews.

The following information was extracted from this subset of 13 review articles into a table for consideration by the working group:

1. Review questions
2. Type of review (e.g., systematic, rapid, realist, etc.)
3. Definition of intervention
4. Definition of scaling up, and
5. General conclusions
6. **Frameworks for Scaling up**

The second search was undertaken to specifically identify frameworks for scaling up in the literature. Guided by the input of the panels, a targeted search was undertaken. This search involved:

- A review of the bibliographies of three key scaling up resources: ExpandNet-WHO toolkit,^14^ a rapid review of ‘large scale change’ by Atkinson et al.,^13^ and a review of scaling-up frameworks by Subramanian et al.^12^;
- A targeted search within the organizational sciences literature (Scopus and Web of Science) using key words: “whole scale change/large scale change” and “framework/model/theory/concept”. This process identified 18 new articles.
- A review of specific sources suggested by panel members (the Spring 2014 Stanford Social Innovation Review journal (focused on scaling up), and Trent & Chavis’ 2009 article on scale, scope, and sustainability^15^).

As in the first search, frameworks identified through this search were reviewed and selected by two analysts for inclusion based on their relevance to complex, multi-component interventions, with a focus on ensuring inclusion of frameworks from across disciplines. This process yielded six frameworks in six documents.

1. ExpandNet framework ^16^
2. Scaling up management (SUM) ^17^
3. Framework for development effectiveness from literature and practice ^18^
4. Framework for successful scale up of health interventions ^19^
5. Alternative strategies for scaling up NGOs ^20^
6. World Bank’s framework for scaling-up impact of good practices in rural development ^21^

From the above frameworks, the following information was abstracted for consideration by the working group:

- Cited theories
- Variables / concepts and relationships among them
- Pathways for scaling up
- Substantive focus
- Sources (since the same framework often appeared in more than one source)

A subset of 12 additional sources was identified from the above search.^15 22-31^ These sources did not contain a specific framework, however were considered by the review team to provide useful information relevant to the task of the working group. The same set of data extracted from the frameworks was also extracted from these articles where possible.

1. **Pathways for scaling up**

The third phase focused on abstracting information specific to mechanisms or pathways for scaling up. This phase did not involve a new search but abstracted different types of information from the documents identified in the first two searches:

1. Definitions of the term “scaling up”;
2. Definitions and descriptions of the different ‘pathways’ for scaling up (e.g., horizontal, vertical, functional scaling up, etc.). Given that the literature does not use a single set of names/categories for pathway types, alternative terms for each pathway were also identified and grouped together when appropriate;
3. Information specific to a) processes underlying each pathway, b) considerations associated with the implementation of each pathway, and c) the execution of two or more pathways concurrently.
4. **Barriers and enablers to scaling up**

The focus of this search was an exploration of the barriers and enablers of scaling up. A recent review of large-scale change in complex health settings (Atkinson et al., 2013) served as a starting point for factors that influence scaling up. Factors were seen as considerations that could either help or inhibit the process of scaling up (e.g., fiscal resources, political support, etc.). Other sources identified as part of the first two searches and an unpublished document identifying considerations for scaling up (provided by the knowledge user panel) were also scanned for barriers/enablers. Similar examples of barriers and enablers were added where there was overlap and new factors were also identified and added to the list. Factors were then organized according to five contextual domains of scale up (i.e. environment, set of interventions, scaling up strategy, user organization, and originating organization).

**Final list of references used to develop scaling up program theory:**

1. Novins DK, Green AE, Legha RK, et al. Dissemination and implementation of evidence-based practices for child and adolescent mental health: a systematic review. Journal of the American Academy of Child and Adolescent Psychiatry 2013;**52**(10):1009-25.e18.

2. Hirschhorn LR, Talbot JR, Irwin AC, et al. From scaling up to sustainability in HIV: potential lessons for moving forward. Globalization and health 2013;**9**:57.

3. LaRocca R, Yost J, Dobbins M, et al. The effectiveness of knowledge translation strategies used in public health: a systematic review. BMC Public Health 2012;**12**:751.

4. Wallin L. Knowledge translation and implementation research in nursing. International journal of nursing studies 2009;**46**(4):576-87.

5. Chaudoir SR, Dugan AG, Barr CH. Measuring factors affecting implementation of health innovations: a systematic review of structural, organizational, provider, patient, and innovation level measures. Implement Sci 2013;**8**:22.

6. Willey BA, Paintain LS, Mangham L, et al. Strategies for delivering insecticide-treated nets at scale for malaria control: a systematic review. Bull World Health Organ 2012;**90**(9):672-84e.

7. Cresswell K, Sheikh A. Organizational issues in the implementation and adoption of health information technology innovations: an interpretative review. International journal of medical informatics 2013;**82**(5):e73-86.

8. Child S, Goodwin V, Garside R, et al. Factors influencing the implementation of fall-prevention programmes: a systematic review and synthesis of qualitative studies. Implement Sci 2012;**7**:91.

9. Semenic S, Childerhose JE, Lauziere J, et al. Barriers, facilitators, and recommendations related to implementing the Baby-Friendly Initiative (BFI): an integrative review. Journal of human lactation : official journal of International Lactation Consultant Association 2012;**28**(3):317-34.

10. Henderson C, Beach A, Finkelstein N. Facilitating Change in Undergraduate STEM Instructional Practices: An Analytic Review of the Literature. Journal of Research in Science Teaching 2011;**48**(4):952-84.

11. Chen PG, Diaz N, Lucas G, et al. Dissemination of results in community-based participatory research. Am J Prev Med 2010;**39**(4):372-8.

12. Subramanian S, Naimoli J, Matsubayashi T, et al. Do we have the right models for scaling up health services to achieve the Millennium Development Goals? BMC Health Serv Res 2011;**11**:336.

13. Atkinson J, Patel C, Wilson A, et al. Drivers of large-scale change in complex health systems: an Evidence Check rapid review. Sydney: Sax Institute for the NSW Agency for Clinical Innovation, 2012.

14. World Health Organization (WHO). Nine Steps for Developing a Scaling‐Up Strategy. Geneva: WHO, 2010.

15. Trent TR, Chavis DM. Scope, Scale, and Sustainability: What It Takes to Create Lasting Community Change. The Foundation Review 2009;**1**(1, Article 8).

16. Simmons RS, Fajans P, Ghiron L. Scaling up health service delivery: from pilot innovations to policies and programmes. Geneva, Switzerland: World Health Organization Press, 2007.

17. Cooley L, Kohl R. Scaling Up—From Vision to Large-scale Change: A Management Framework for Practitioners. Washington DC: Management Systems International, 2006.

18. Hartmann A, Linn J. Scaling up: A framework and lessons for development effectiveness from literature and practice. Wolfensohn Center for Development, Working Paper 5. Washington: The Brookings Institution, 2008.

19. Yamey G. Scaling up global health interventions: a proposed framework for success. PLoS Med 2011;**8**(6):e1001049.

20. Uvin P. Fighting hunger at the grassroots: Paths to scaling up. World Development 1995;**23**(6):927-39.

21. World Bank. Scaling Up the Impact of Good Practices in Rural Development Washington D.C.: Agriculture & Rural Development Department, World Bank, 2003.

22. Berwick DM. Disseminating innovations in health care. Jama 2003;**289**(15):1969-75.

23. Paina L, Peters DH. Understanding pathways for scaling up health services through the lens of complex adaptive systems. Health Policy Plan 2012;**27**(5):365-73.

24. Binswanger H, Nguyen TV. A step by step guide to scale up Community Driven Development. International workshop on ‘African Water Laws: Plural Legislative Frameworks for Rural Water Management in Africa, 26-28 January 2005. Johannesburg, South Africa, 2005.

25. Anderson I. Scaling Up Development Results: A Literature Review and Implications for Australia’s Aid Program. Canberra: Office of Development Effectiveness, Australian Agency for International Development (AusAID), 2012.

26. Gonsalves J. Going to scale: Can we bring more benefits to more people more quickly? Workshop highlights 10-14 April, IIRR , Philippines. Philippines: CGIAR-NGO Committee and The Global Forum for Agricultural Research with BMZ (German Ministry of Development Corporation), MISEREOR (German Catholic Church Development Agency), Rockefeller Foundation, IRRI (International Rice Research Institute), and IIRR (International Institute of Rural Reconstruction). 2000.

27. Foster-Fishman PG, Watson ER. The ABLe change framework: a conceptual and methodological tool for promoting systems change. Am J Community Psychol 2012;**49**(3-4):503-16.

28. Söderlund J. Knowledge entrainment and project management: The case of large-scale transformation projects. International Journal of Project Management 2010;**28**(2):130-41.

29. Bradach J, Grindle A. Transformative scale: the future of growing what works. Stanford Social Innovation Review 2014;**Spring 2014**.

30. Bartczak L. Leveraging a Movement Moment. Stanford Social Innovation Review 2014;**Spring 2014**.

31. Enright K. What would it take? . Stanford Social Innovation Review 2014;**Spring 2014**.
